# Stratifying the shoreline: a modified OSPAR framework to monitor event-driven beach litter

**DOI:** 10.1007/s10661-026-15260-x

**Published:** 2026-04-10

**Authors:** Zara Teixeira, Ana Cunha, Paula C. S. Carvalho, Carlos Gonçalves, Cátia Marques, A. Cristina Rocha, Ana Bóia, Diana Pacheco, Diana Marques, Luís Resende, João Fernandes, Cláudia Moreira

**Affiliations:** 1 MARE – Marine and Environmental Sciences Centre, ARNET – Aquatic Research Network, Department of Life Sciences, University of Coimbra, Colégio de S. Bento, Calçada Martim de Freitas, 3000-456 Coimbra, Portugal; 2 MARE – Marine and Environmental Sciences Centre, Department of Life Sciences, University of Coimbra, Colégio de S. Bento, Calçada Martim de Freitas, 3000-456 Coimbra, Portugal; 3 GeoBioTec-GeoBioSciences, GeoTecnhologies and GeoEngineering Research Centre, Department of Geosciences, Campus de Santiago, University of Aveiro, 3810-193 Aveiro, Portugal; 4 MARE – Marine and Environmental Sciences Centre, ARNET – Aquatic Research Network, Instituto de Investigação e Formação Avançada (IIFA), Palácio Do Vimioso, Largo Marquês de Marialva, Apart. 94, 7002 - 554 Évora, Portugal

**Keywords:** Marine litter monitoring, Stratified sampling, OSPAR methodology, Coastal events, Single-use plastics, Beach pollution hotspots

## Abstract

Urban beaches are increasingly vulnerable to litter accumulation, especially during large-scale coastal events that create short-lived but intense pollution pulses. Despite growing interest in marine litter monitoring, traditional methods often lack the spatial and temporal sensitivity required to capture such episodic surges. This study presents a methodological adaptation of the OSPAR beach litter monitoring protocol, applying a stratified sampling framework to a high-use coastal site during the RFM SOMNII festival in Figueira da Foz, Portugal, one of Europe’s largest beach music festivals. Over a 5-year period (2019–2023), including pre- and post-COVID-19 seasons, 17 seasonal surveys were conducted across three functional zones (STAGE, VIP, CHILLOUT) to assess the spatiotemporal dynamics of litter accumulation. Results indicate clear spatial heterogeneity, with litter densities peaking in high-traffic areas and artificial polymer materials, particularly single-use plastics, accounting for over 90% of all litter items. Temporal trends show sharp declines in 2020–2021 during festival cancellations, with subsequent rebounds following the event’s return, and further reductions after targeted cleanup measures in 2023. The stratified sampling approach revealed patterns and hotspots that would likely be overlooked by conventional OSPAR layouts, highlighting the potential for this framework to enhance marine litter monitoring in event-prone coastal zones. Findings also inform broader sustainability strategies, reinforcing the need for adaptive cleanup planning, reusable alternatives to single-use items, and coordinated engagement between researchers, event organizers, and policymakers. The approach offers a replicable blueprint for improving beach litter assessments under dynamic, high-pressure conditions worldwide.

**Supplementary Information:**

The online version contains supplementary material available at 10.1007/s10661-026-15260-x.

## Introduction

Marine litter represents a pressing global environmental challenge with broad implications for marine ecosystems, human health, and local economies. Plastics dominate this pollution stream and accumulate persistently on shorelines (Garcés-Ordóñez et al., 2020; Grelaud & Ziveri, 2020), where they reduce aesthetic value, threaten wildlife, and interfere with human activities (Browne et al., 2015). Although only around 0.1% of global plastic production is estimated to enter the ocean annually (Cózar et al., 2014), this still represented between 5.5 and 14.5 million metric tons in 2018 (Wayman & Niemann, 2021), highlighting the scale and persistence of the problem.

Recent regional assessments across the OSPAR North-East Atlantic reveal persistent and substantial beach litter pollution. Median litter abundances between 2018 and 2020 reached levels an order of magnitude above the European Threshold Value for Good Environmental Status, with typical counts around 252 items per 100 m compared to the recommended 20 items per 100 m (Lacroix et al., 2022; van Loon et al., 2020). Plastics dominate the region’s beach litter, comprising 94% of all recorded items. Among these, single-use plastics (SUP) and maritime-related items (SEA), both addressed by the EU Single-Use Plastics Directive (Directive 2019/904), contribute around 45 and 36 items per 100 m, respectively. These regional trends are also evident at finer spatial scales. Along the North Atlantic Iberian coast, long-term OSPAR monitoring (2002 –2020) reveals comparable patterns, with plastics consistently representing the majority of beach litter (around 70%) and fishing-related items contributing a further 15% (Andriolo & Gonçalves, 2025). Over the past two decades, litter abundances have ranged from 90 to 259 items per 100 m, with a gradual upward trend of roughly 9 items per 100 m per year. Within this subregion, beaches in the Lisbon and Central areas of Portugal have repeatedly shown the highest pollution levels. These findings highlight the chronic nature of beach litter pollution on the Iberian Coast and reinforce the need for monitoring approaches capable of capturing both widespread, recurrent items and short-lived but intense inputs.

At the local scale, these regional and subregional patterns manifest most strongly on urban beaches. Their accessibility, popularity among tourists, and high foot traffic contribute to higher litter loads (Garcés-Ordóñez et al., 2020; Grelaud & Ziveri, 2020). As dynamic interfaces between terrestrial and marine systems, beaches act both as sinks and sources of anthropogenic debris (Gallitelli et al., 2023; Jambeck et al., 2015), making beach litter one of the most visible indicators of environmental pollution. Beyond their role as persistent accumulation zones, beaches exhibit strong temporal variability. Seasonal fluctuations in litter abundance have been widely documented across the globe, from Spain and China to Brazil and the Mediterranean (Ali & Shams, 2015; Asensio-Montesinos et al., 2019; Grelaud & Ziveri, 2020; Pervez et al., 2020; Ribeiro et al., 2021). In addition to these predictable seasonal patterns, large coastal events can trigger episodic surges in litter deposition, the impacts of which remain poorly characterized and often underestimated (Oliveira et al., 2022). Although such events promote cultural and recreational engagement, they generate substantial waste that, if not effectively managed, may contribute to long-term environmental degradation (Browne et al., 2015).

Although these dynamics are increasingly well documented, a key methodological gap remains in understanding how event-driven litter deposition unfolds across space and time. Traditional beach monitoring protocols, though standardized and robust for long-term trends (e.g. OSPAR Commission, 2022), often lack the spatial resolution and temporal sensitivity needed to detect short-lived yet intense pollution episodes. Overlooking these episodic surges risks underestimating total litter inputs (Sodré et al., 2025), misattributing pollution sources, and neglecting localized hotspots that pose disproportionate ecological and management challenges (Browne et al., 2015). In this context, methodological innovations are essential to develop monitoring strategies capable of capturing dynamic littering phenomena with sufficient granularity.

This study addresses that gap by adapting the OSPAR methodology to a stratified sampling framework tailored to high-traffic, event-prone coastal areas. Specifically, the research applies this design to the case of RFM SOMNII, one of Europe’s largest beach music festivals, held annually in Figueira da Foz, Portugal. With over 100,000 participants each year (RFM SOMNII Festival, 2025), this site offers a valuable testbed to examine the performance and utility of stratified monitoring in capturing event-driven litter fluctuations.

The objective of this paper is to assess the performance of this adapted monitoring design through a 5-year dataset encompassing seasonal surveys at Praia do Relógio (Central Region of Portugal), spanning pre- and post-COVID-19 periods. By examining litter abundance, composition, and spatial distribution, the study aims to demonstrate how stratified OSPAR-based protocols can improve the characterization of marine litter patterns associated with large-scale events, contribute to the refinement of international monitoring frameworks, and support more adaptive coastal management strategies.

## Methodology

### Study site and beach music festival

**Fig. 1:**
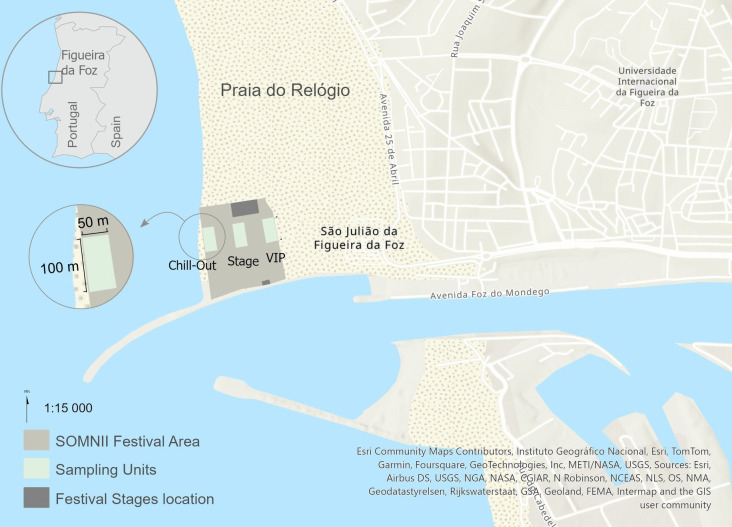
Location of the RFM SOMNII Beach Music Festival at Praia do Relógio, Figueira da Foz, Portugal, showing the stratified sampling units classified as VIP, STAGE, and CHILLOUT zones. Each sampling unit measures 100 m × 50 m

The study was conducted at Praia do Relógio, an urban beach in Figueira da Foz (40° 149,161 N, 8° 871,150 W), on the North Atlantic Portuguese coast (Fig. 1). The beach has an average width of 500 m and extends approximately 2 km in a North-South orientation. The city experiences high levels of tourism during the summer months (mainly July and August), and the beach is public and freely accessible. However, the sampling area is not as popular with sunbathers compared to other areas at the same beach, likely due to a combination of three factors: the beach’s large width, its southern boundary being marked by a jetty (Lira et al., 2016), and the western boundary being limited by dunes (Andriolo & Gonçalves, 2023), which restrict direct access to the area.

The sampling area is located at the site of the RFM SOMNII Beach Music Festival (RFM SOMNII Festival, 2025), which held its first edition in 2013. Since then, the festival has taken place annually at the beginning of July (summer season), except for the years 2020 and 2021, when it was cancelled due to the COVID-19 pandemic. This festival, recognized as the largest beach music festival in Portugal and one of the biggest in Europe, typically attracts around 100,000 attendees for 72 h of music during the afternoons and nights. In 2023, the SOMNII producers organized a second festival, BR FEST, just 1 week later, at the same location and using the same infrastructure. BR FEST was described as the “biggest ever event dedicated to Brazilian music in Portugal,” with “thousands” of participants (BR FEST 2023, n.d.).

### Field sampling design

The OSPAR guidelines for monitoring marine litter on beaches (Wenneker et al., 2010) specify a standard sampling unit of 100 m, measured as a straight line parallel to the back of the beach. However, while OSPAR guidelines cover the area between the swash zone and the backshore, this study defined three parallel sampling units, each measuring 100 m × 50 m, within the festival area (total festival area = 110 000 m^2^) (Fig. 1). This adapted methodology was necessary to implement a stratified sampling design representative of the main festival areas: the VIP zone, the STAGE zone, and the CHILLOUT zone. The VIP zone featured a carpeted floor and was located on the east side of the enclosure, at the backshore. The STAGE zone was the middle area where a large number of visitors congregated for long periods. The CHILLOUT zone was located between the food and drink tents and the swash zone, and was mainly used for resting during the festival (https://vimeo.com/rfmsomnii).

**Fig. 2:**
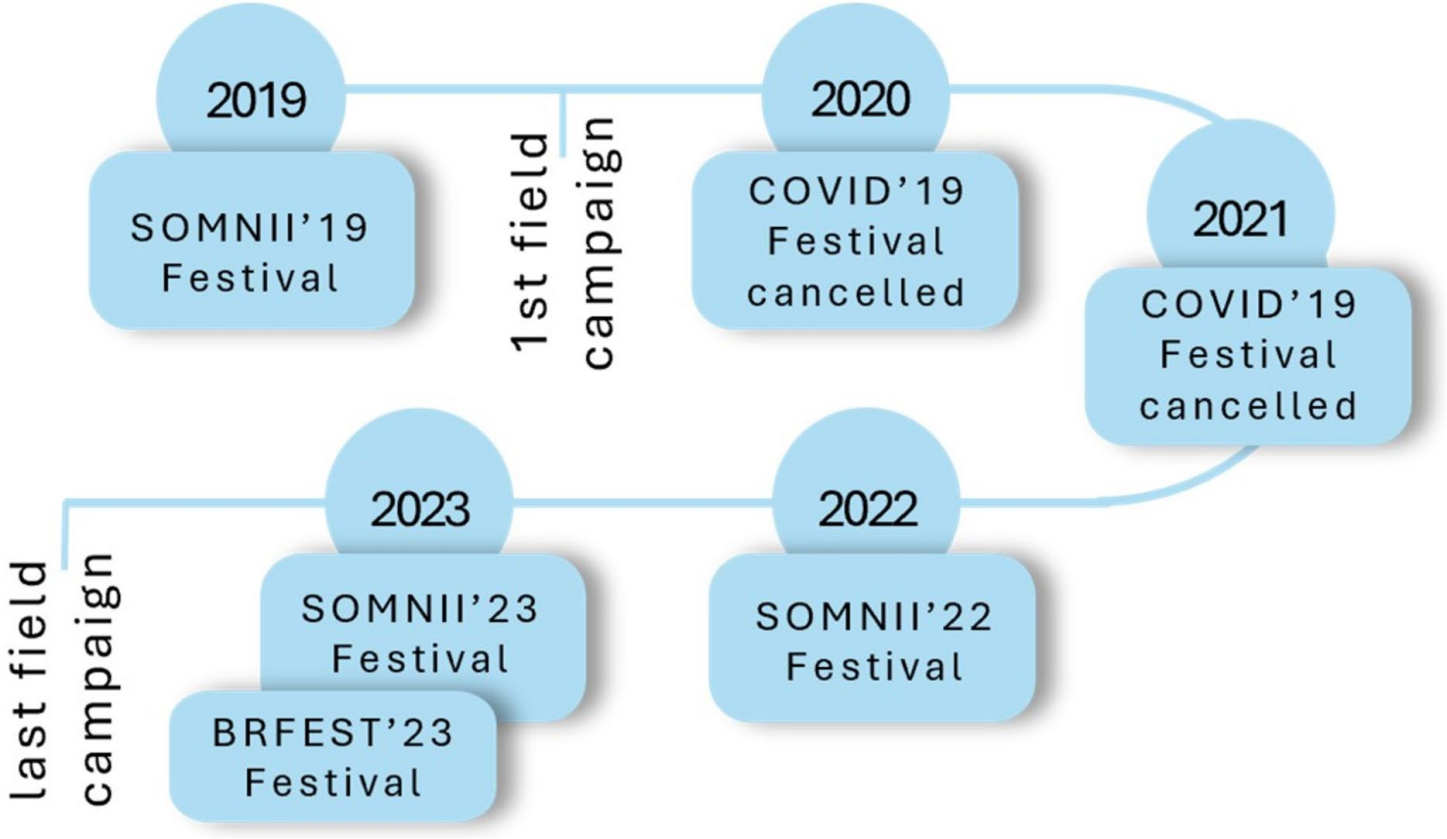
Timeline illustrating key events in the history of the RFM SOMNII festival and related occurrences between 2019 and 2023. The timeline highlights significant milestones, including the cancellation of the festival in 2020 and 2021 due to the COVID-19 pandemic and the first and last field campaigns conducted after SOMNII’19 and BR FEST’23. BR FEST’23 was a second festival held at the same location, using the same infrastructure. A total of 17 seasonal sampling campaigns were conducted between summer 2019 and summer 2023. Immediate post-festival field campaigns were conducted one to two weeks after the complete dismantling of the infrastructure

Field campaigns were conducted between summer 2019, shortly after the SOMNII festival of 2019, and summer 2023, following the 2023 edition of the festival (Fig. 2). In total, 17 seasonal campaigns were carried out. The first two took place in 2019 (summer and autumn). Four campaigns were conducted in each of the years 2020, 2021, and 2022 (winter, spring, summer, autumn). Finally, three campaigns were completed in 2023 (winter, spring, summer), with the last one coinciding with the post-festival period in summer 2023. All sampling was conducted only after the festival infrastructure had been fully removed, ensuring that surveys reflected beach conditions without temporary structures. Each campaign began approximately 2 h before low tide, with two to three individuals surveying each designated unit (VIP, STAGE, CHILLOUT) along transects.

### Macro-litter sampling and identification

On average, 1 to 2 months after sampling, beach litter was sorted, counted, measured (when relevant), and assigned to one of the 112 predefined OSPAR litter types (Wenneker et al., 2010). The latest OSPAR guidelines classify cigarette butts under the artificial polymer material category, aligning with their plastic composition. As a result, we did not adopt the approach used in previous studies (Araújo & Costa, 2021; Bettencourt et al., 2023), where cigarette butts were categorized as a separate group. Those studies relied on earlier OSPAR guidelines that inaccurately placed cigarette butts in the paper/cardboard category, necessitating a workaround to account for their unique nature.

Four individuals participated in the categorization process, with the project coordinator supervising to ensure consistent decision-making criteria for ambiguous items. All items were classified according to the following criteria:

- Material composition, as defined in the MSFD recommendations (MSFD Technical Subgroup on Marine Litter, 2013): artificial polymer material (also known as plastic), rubber, cloth/textile, paper/cardboard, processed/worked wood, metal, glass/ceramics, undefined.
- Single-use plastics (SUP) and maritime-related plastics (SEA): The SUP category follows the definitions in the MSFD recommendations (MSFD Technical Subgroup on Marine Litter, 2013). The SEA category is derived from the FISH category in the MSFD recommendations (Hanke et al., 2019), excluding non-plastic items.
- Litter types targeted by existing measures: Specifically, items addressed in the OSPAR Marine Litter Regional Action Plan (OSPAR Commission, 2022) or the EU SUP Directive (Directive 2019/904).
- SOMNII items and OTHER items: This classification is based on the presumed source of the items. SOMNII items are those confidently identified as left behind by festival participants or staff, such as plastic cups with the festival logo, festival tickets, bracelets and zip ties (Supplementary Material - Images). Cigarette butts were also included in the SOMNII category due to their significant presence during post-festival sampling, especially in the STAGE zone, where the highest attendee concentration is observed (Fig. 4). OTHER items include all remaining litter items not linked to the festival or whose source could not be determined.

### Data analysis

The Pearson’s chi-square test of independence was initially performed to evaluate the association between litter categories and the predictor variables: ‘source,’ ‘sampling unit,’ ‘season,’ and ‘year.’ This non-parametric test was selected due to its appropriateness for categorical data and its ability to assess whether observed frequencies significantly deviate from expected frequencies. The test was performed at a 5% significance level, and the results were interpreted based on the calculated chi-square values and their corresponding *p*-values. The chi-test was carried out using the R Stats package (R Core Team and contributors worldwide, 2025).

Abundances were assessed by calculating the median of items at the zone scale (for each sampling unit) and aggregated at the site scale (total sampling area) (Table 1). Median-based statistical methods were chosen because they are well suited for handling skewed distributions in beach litter data (Schulz et al., 2017, 2019). It is worth noting that median values are typically lower than mean (average) values, as they exclude extreme outliers, making them more appropriate for decision-making purposes. To calculate percentages, the median of each litter group was divided by the total sum of the medians across all litter groups considered (e.g., the percentage of artificial polymer material is obtained by dividing its median by the total sum of the medians of all material categories). The top 15 litter types were ranked based on the median values of individual litter types. Trends were assessed using the median-based Theil-Sen method, that calculates slopes and associated p-value. Analyses were performed using the litteR package (Walvoort & van Loon, 2021) and Microsoft Excel.

**Table 1:** List of statistical indicators calculated at the zones and site geographical scales and corresponding calculation methods

Beach litter indicator	SOMNII zones scale		SOMNII site scale
Abundance	Median of sampling unit data for the four seasons sampled, within the five-year period from Summer 2019 to Summer 2023		Median of sampling units’ medians
Percentage	100 × median of the category considered divided by the sum of medians of the different categories		
Top 15	Ranking of medians of individual litter types and presentation of the 15 highest ranking types		
Trend	Theil-Sen slope of sampling unit data for the five-year period from 2019 to 2023. *p*-value of Theil-Sen slope	Median of sampling units’ slopes. *p*-value of aggregated sampling units	

The relationship between the number of items collected per litter type and the predictor variables, ‘category,’ ‘source,’ ‘sampling unit,’ ‘season,’ and ‘year,’ was explored to identify the predictors that most strongly influence the number of items collected.

**Fig. 3:**
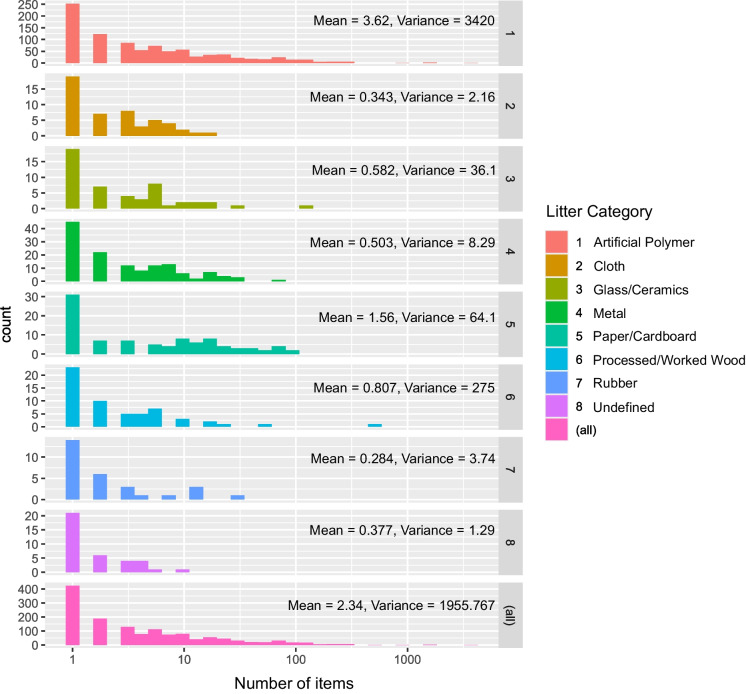
Histograms illustrating the frequency distribution of litter items by material composition: artificial polymer materials (plastic), cloth, glass/ceramics, metal, paper (paper/cardboard), processed/worked wood, rubber, and undefined. Mean and variance values are provided for each category, highlighting the variability in item counts. Artificial polymer materials exhibit the highest mean and variance

Preliminary analysis revealed data overdispersion (variance significantly higher than the mean) (Fig. 3) indicating the need for a Negative Binomial distribution model. Additionally, it was assumed that the dataset includes structural count zeros arising from two distinct processes: sampled areas that were cleaned beforehand and litter types deemed irrelevant to the festival context. As such, a Zero-Inflated Negative Binomial (ZINB) model was implemented, using the pscl package from R software (Jackman, 2024; Zeileis et al., 2008). The ZINB model assumes that excess zeros arise from a separate process, independent of the count data, and models these zeros separately. It consists of two components: the logit model to predict the “certain zero” cases, identifying whether a variable belongs to this group; and a Negative Binomial model to predict counts for data not classified as “certain zeros.” In this study, particular interest lies in understanding which variables are predictors of the excess zeros, to support the hypotheses that certain items are absent because they are not from SOMNII sources. In other words, during festival years, certain items are highly more expected than others.

All statistical analyses were performed using R Software version 4.4.2.

## Results

### Spatial and temporal distribution of beach litter

**Fig. 4:**
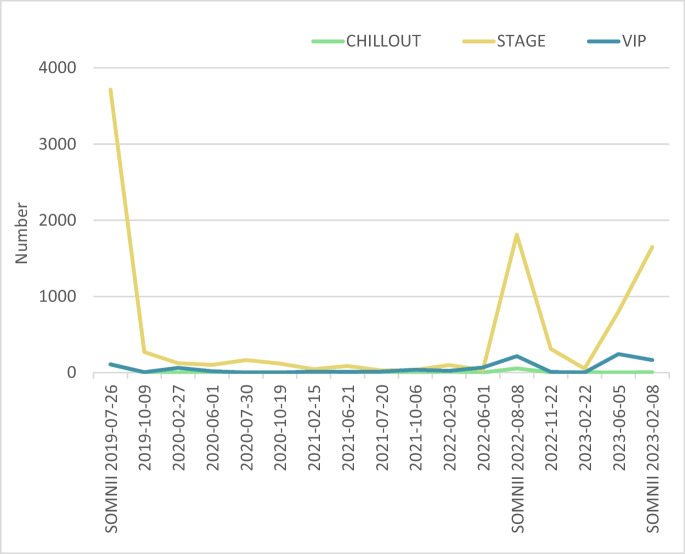
Temporal variation in cigarette butt accumulation shown by festival zone (VIP, STAGE, and CHILLOUT). Peaks in litter counts are observed in the STAGE zone during surveys conducted immediately after festival editions, whereas the CHILLOUT and VIP zones show relatively stable and consistently lower counts of cigarette butts across all sampling dates

Exploratory analysis showed that cigarette butts were highly prevalent in post-festival samples, especially in the STAGE zone, where attendee density was highest (Fig. 4). Although their exact origin cannot be confirmed, their strong spatial variability and concentration in this zone support their classification within the SOMNII category, and all subsequent analyses follow this classification (Fig. 4).

Statistically significant associations (*p* < 0.05) were found between litter categories and their source, sampling units, seasons, and years, indicating that litter composition varied meaningfully with these factors (Table 2) (Supplementary Material_Chi-SquareTest).

**Table 2:** Pearson’s chi-squared test of independence to evaluate the association between litter categories and the categorical variables source (SOMNII/OTHER), sampling unit (VIP, STAGE, and CHILLOUT zones), sampling season (winter, spring, summer, autumn), and sampling year (2019, 2020, 2021, 2022, 2023)

	Chi-square value	df	*p*-value
Litter categories VS source	4075.6	7	< 2.2e−16 *
Litter categories VS sampling unit	655.06	14	< 2.2e−16 *
Litter categories VS season	1052.9	21	< 2.2e−16 *
Litter categories VS year	1358.5	28	< 2.2e−16 *

*p-value < 0.05 (statistically significant)

A total of 26,742 items were collected in the SOMNII festival sampling site from 2019 to 2023, with a total count median of 329 items/100 m. Overall, the median is lower for items that have a SOMNII source (94 items/100 m) compared to those from OTHER sources (235 items/100 m). Median item densities were highest in the STAGE zone (532 items/100 m), followed by the VIP zone (329 items/100 m), and lowest in the CHILLOUT zone (70 items/100 m). Seasonally, median densities peaked in summer (569 items/100 m) and were lowest in winter (184 items/100 m).

**Fig. 5:**
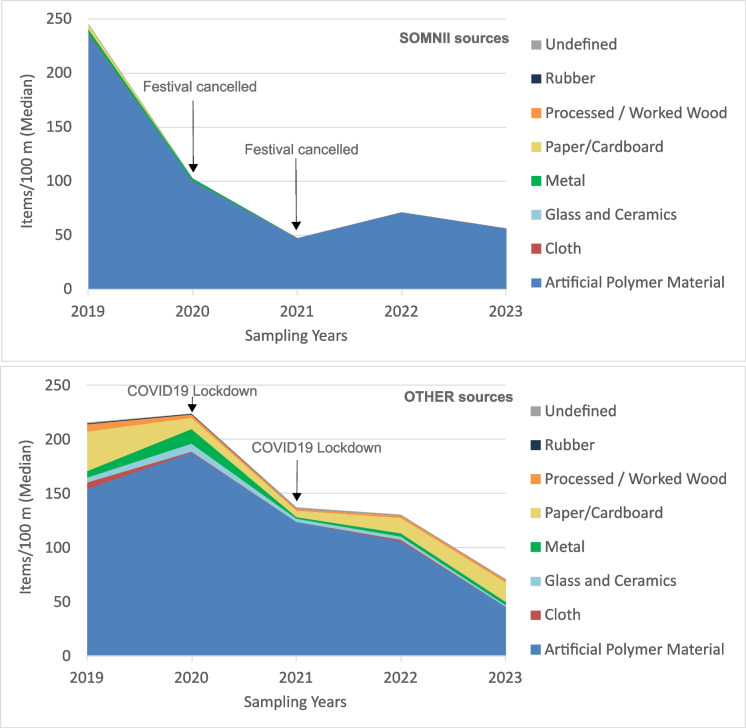
Trends in the median number of litter items per 100 m across all surveys (17 campaigns) and all years (2019–2023), shown by litter category, and source type (“SOMNII sources” at the top and “OTHER sources” at the bottom). From SOMNI sources, the median values for all categories remained at 0 across all years, except for artificial polymer materials, paper/cardboard, and metal

The magnitude of beach litter pollution from SOMNII sources shows a different trend over the years compared to OTHER sources. Items from SOMNII sources showed a sharp decrease between 2019 and 2021, a time period without festival editions that covers the COVID-19 pandemic, followed by an increase in 2022 when the festival returned, and a subsequent decrease in 2023 when massive cleanups were implemented by the festival organization (Fig. 5). Despite the trends observed, no significant decreases in both total counts and plastic items have been observed for items from SOMNII sources, at the site and at the zone scales (Table 3). Items from OTHER sources experienced an increase between 2019 and 2020, before the COVID-19 lockdown, followed by a sharp decrease between 2020 and 2021 (COVID-19), a slight decrease until 2022 (after COVID-19), and another sharp decrease until 2023 (massive cleanups related to the SOMNII festival) (Fig. 5).

**Table 3:** Median total and plastic counts (from 2019 to 2023), and associated trends, for items from SOMNII sources, in the site area and zones

		TOTAL			PLASTIC (artificial polymer materials)		
Geographical scale		Median count (items/100 m)	Trend (slope in items/100 m per year)	*p*-value	Median count (items/100 m)	Trend (slope in items/100 m per year)	*p*-value
Site area		329	−24.46	0.0875	282	−36.42	0.0875
Zones	VIP	329	−24.46	0.2712	282	−36.42	0.3575
	STAGE	532	−86.12	0.2167	457	−80.55	0.2448
	CHILLOUT	70	−10.97	0.1715	56	−10.8	0.1006

All *p*-values are not significant

**Fig. 6:**
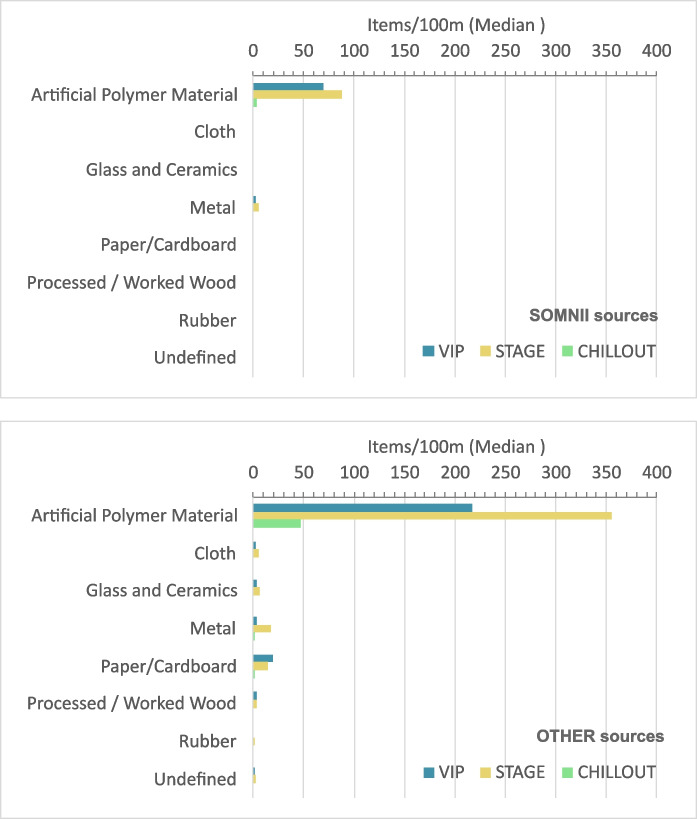
Median number of litter items per 100 m across all surveys (17 campaigns) and all years (2019–2023), shown by litter category, festival zone (VIP, STAGE, CHILLOUT), and source type (“SOMNII sources” at the top and “OTHER sources” at the bottom). Artificial polymer materials are the dominant category overall. Among the items confidently attributed to the SOMNII festival, counts are highest in the STAGE zone, whereas plastic items from other sources reach their highest values in the VIP zone

The analysis of litter composition shows a predominance of items made of artificial polymer materials, also known as plastics (Fig. 6). At the site scale (SOMNII Festival area scale), plastic items represent 90.38% of the pollution, with a median of 282 items/100 m whereas other materials do not exceed 14 items/100 m. Similar results are observed at the VIP (88.68%), the STAGE (89.78%), and the CHILLOUT (96.55%) zones.

When analyzing litter composition by source, the medians indicate that items from SOMNI sources belong only to four categories: artificial polymer materials (including reusable plastic cups, zip ties, beverage-related items, food wraps, plastic pieces), metal (including wire and containers), paper/cardboard, and cloth (Fig. 6). Artificial polymer materials are the dominant category overall. Among the items we can confidently attribute to the SOMNII festival (SOMNII sources), the highest counts occur in the STAGE zone, where most participants gather during the event. The remaining plastic items show the highest counts in the VIP zone, which is farther away from the swash zone and closer to the dunes. Top litter types are discussed in more detail in another section.

### Predicting the number of items

There is a statistically significant probability that all category items, except for paper/cardboard, appear less frequently than artificial polymer materials (Table 4). For instance, cloth items are 1.67 times less likely to appear than artificial polymer items. Items from the SOMNII festival are 1.05 times more likely to appear than items from OTHER sources. This does not imply a greater number of SOMNII items overall, but rather that these items have a higher probability of occurrence within a specific category and zone. Additionally, SOMNII items are 0.76 times more likely to be collected in the STAGE zone than in the VIP zone, but 1.16 times less likely to be collected in the CHILLOUT zone. This is in line with the intensity of festival goers expected in each of three sampling units.

**Table 4:** Zero-inflated model fitted to the variables “category,” “source,” and “sampling unit” to model the count in the negative binomial part. The variables “category,” “source,” “sampling unit,” and “year” model the logit part. This model fits the data significantly better than the null model (intercept-only), with a *p*-value of 8.937496e−232 (df = 27)

Model formula: nr ~ cat + source + samplingunits | source + samplingunits + year + season					
Count model (negative binomial) coefficients					
	Estimate	Std. Error	*z* value	Pr(>|*z*|)	
(Intercept)	1.00874	0.09641	10.463	< 2.00E−16	***
Cloth	−1.66619	0.26623	−6.258	3.89E−10	***
Glass and ceramics	−1.21497	0.24856	−4.888	1.02E−06	***
Metal	−1.64681	0.1612	−10.216	0.65759	***
Paper/cardboard	−0.08986	0.20273	−0.443	0.01721	
Processed/worked wood	−0.67395	0.28291	−2.382	1.28E−12	*
Rubber	−2.17006	0.30578	−7.097	0.00126	***
Undefined	−1.36214	0.42241	−3.225	3.16E−11	**
SOMNII source	1.04711	0.15773	6.639	3.11E−09	***
STAGE	0.76317	0.12879	5.926	< 2.00E−16	***
CHILLOUT	−1.15969	0.12766	−9.084	< 2.00E−16	***
Log(theta)	−2.74007	0.03893	−70.391	< 2.00E−16	***
Zero-inflation model (logit) coefficients					
	Logg−odds	Std. Error	*z* value	Pr(>|*z*|)	
(Intercept)	−45.5334	5.71E+04	−0.001	0.99936	
Cloth	1.3557	4.94E−01	2.747	0.00601	**
Glass and ceramics	17.4494	1.03E+03	0.017	0.98649	
Metal	0.6282	2.57E−01	2.442	0.01462	*
Paper/Cardboard	1.3063	3.30E−01	3.956	7.61E−05	***
Processed/worked wood	43.2256	5.71E+04	0.001	0.9994	
Rubber	1.7047	6.63E−01	2.573	0.01009	*
Undefined	19.0714	4.09E+03	0.005	0.99628	
SOMNII source	45.0296	5.71E+04	0.001	0.99937	
STAGE zone	0.1627	1.82E−01	0.896	0.37009	
CHILLOUT zone	1.1992	2.21E−01	5.429	5.67E−08	***
2020	0.8892	2.96E−01	3.003	0.00267	**
2021	1.1892	3.00E−01	3.96	7.48E−05	***
2022	0.8084	2.96E−01	2.734	0.00626	**
2023	0.9713	3.12E−01	3.116	0.00183	**

Signif. codes: 0 “***” 0.001 “**” 0.01 “*” 0.05 “.” 0.1 “ ” 1

Theta = 0.0622. Number of iterations = 67. Log-likelihood: −7989(df = 21)

The zero-inflation part of the model indicates that some litter categories (e.g., cloth), the zone and the years, have a meaningful impact on the probability of an observation being an excess zero. An increase in the predictor variable (positive estimates) is associated with a higher probability of an observation being an excess zero. In other words, and for the specific case of this study, there is a statistically significant lower probability of collecting cloth, metal, paper, and rubber items. This information is consistent with the fact that most of the items are artificial polymers. In addition, for each item found there is a significant lower probability of collecting items from the CHILLOUT zone than from the VIP zone, suggesting several items were actually more frequently released in this zone. This is in accordance with what we know from the festival SOMNII, that the CHILLOUT zone had the lower number of festival goers and hence, less litter. Finally, there is a significant lower probability of collecting items in 2020 up to 2023 than in 2019, which is again consistent with known events that may result in less litter: festival cancellation, COVID19 and massive cleanups, after 2019.

### Maritime-related plastic items, single-use plastics (SUP), and measures of coverage

The “Maritime-related plastic items” (SEA) and the “Single-use Plastics” (SUP) categories represent 39.46% of litter items.

SEA materials represent 1.08% of litter items and, as expected, do not have a SOMNII source. Their presence is higher in the CHILLOUT zone (2.56%). These results were expected since this sampling unit is closer to the seashore where fishing-related items such as lobster/fish tags, octopus pots, nets, and strings and cords (which can come from different sources but are often mainly related to fishing) are potentially more prone to being found (Ouyang & Yang, 2022; Watson et al., 2022).

**Fig. 7:**
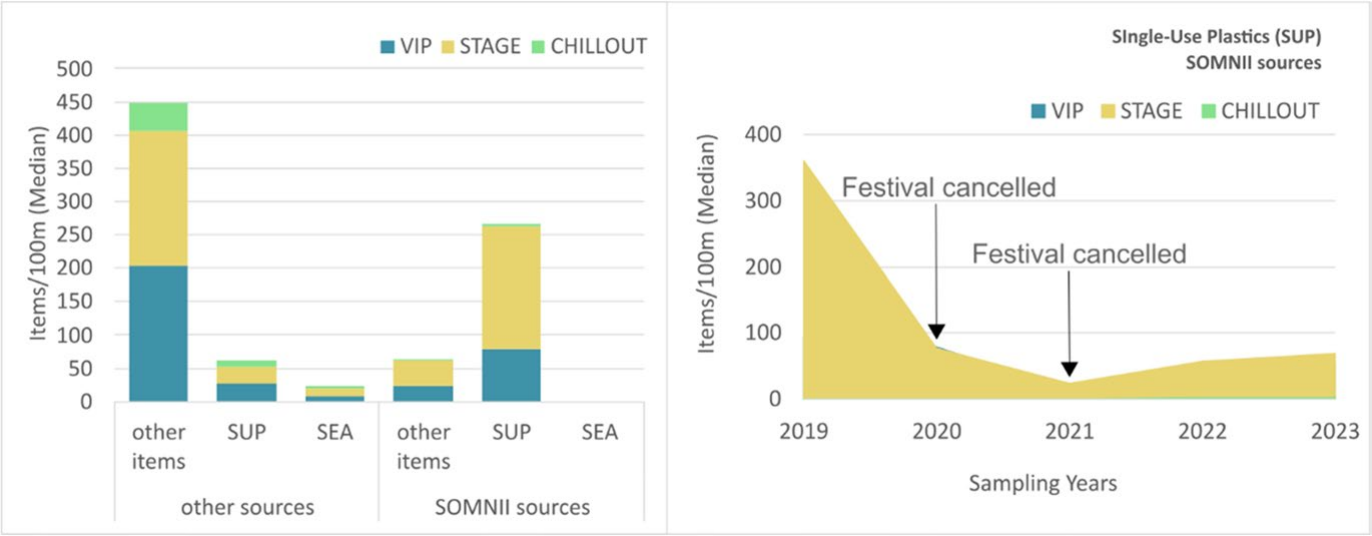
Bar chart (left): median number of litter items per 100 m across all surveys (17 campaigns) and all years (2019–2023), shown by festival zone (VIP, STAGE, CHILLOUT) and source type (“SOMNII sources,” “OTHER sources”), and further categorized by item type (single-use plastics (SUP), maritime-related plastic items (SEA), and other). SUP medians from the SOMNII sources are higher than from OTHER sources and SEA items are absent. Line graph (right): trends in single-use plastic (SUP) items per 100 m from SOMNII sources across yearly surveys, shown by festival zone (VIP, STAGE, CHILLOUT) and sampling year. SUP median counts decline sharply in 2020 and 2021, reflecting festival cancellations related to COVID-19 pandemic

SUP materials represent 38.38% of litter items, 17.54% being attributed to the RFM SOMNII Festival. Median counts were higher at the STAGE zone (183 items/100 m from SOMNII festival and 25 items/100 m from OTHER sources) (Fig. 7). The trend of SUP from the SOMNII source over the years (Fig. 7) follows the same general trend (Fig. 5), indicating a correct classification of these items. However, the trend over the years of both SUP and Other items, from OTHER sources, with increasing trends once the festival returns in 2022, indicates that SOMNII items are potentially sub-accounted.

Fifty precent of the litter items collected are targeted either by the OSPAR Regional Action Plan on Marine Litter (ML RAP) 2014–2020 or the EU SUP Directive (Directive 2019/904).

### Top litter types

**Fig. 8:**
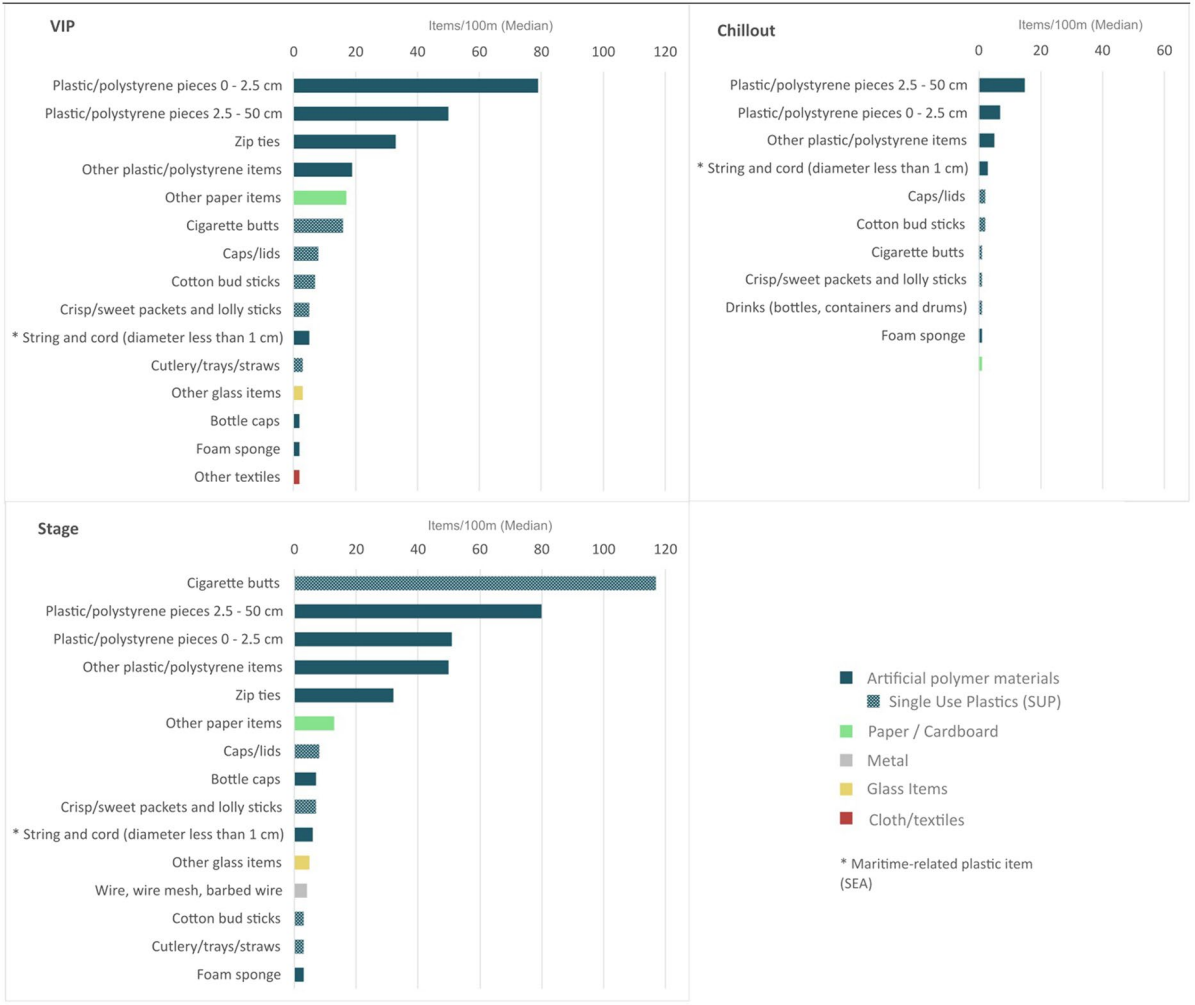
Median number of top 15 litter types (items/100 m) across all surveys (17 campaigns) and all years (2019–2023), shown by festival zone (VIP, STAGE, and CHILLOUT). The VIP zone shows high prevalence of small plastic/polystyrene pieces (0–2.5 cm), the CHILLOUT zone shows a large median number of plastic/polystyrene pieces (2.5–50 cm), and the STAGE zone features a high median number of cigarette butts

The top 15 litter types are dominated by non-identifiable items, such as plastic/polystyrene pieces (0–2.5 cm and 2.5–50 cm) and other plastic/polystyrene fragments, with median values exceeding 50 litter items per 100 m at the VIP and STAGE zones (Fig. 8). These, along with string and cord (diameter less than 1 cm), consist of artificial polymer fragments with untraceable sources. Although some pieces are likely related to fishing-net repair, the presence of these items suggests a potential underestimation of the median of items originating from SOMNII sources.

**Fig. 9:**
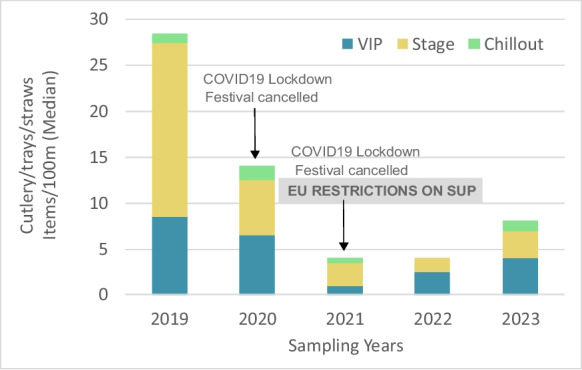
Median number of cutlery/trays/straws per 100 m across yearly surveys, shown by festival zone (VIP, STAGE, CHILLOUT) and sampling year. The declines observed in 2020 and 2021 reflect COVID-19 lockdowns and festival cancellations, as well as the potential influence of EU single-use plastic (SUP) restrictions introduced in 2021

Among the top 15 litter types, six are single-use plastics (SUP) (Directive 2019/904): cigarette butts, caps/lids, cotton bud sticks, crisp/sweet packets and lolly sticks, cutlery/trays/straws, and drink-related items (e.g., bottles). The median for cigarette butts stands out at 117 items per 100 m in the STAGE zone, the area with the highest concentration of festival goers. Other SUP items, such as caps/lids, crisp/sweet packets and lolly sticks, cutlery/trays/straws, and drink-related items, are typical of these types of events. Drink-related items rank among the top 15 in the CHILLOUT zone, whereas cutlery/trays/straws appear in the top 15 only within the VIP and STAGE zones, which is logical given their proximity to meal areas. Given the European ban on plastic plates, cutlery, straws, drink stirrers and containers made of expanded polystyrene, in July 2021 (Currie et al., 2023; Souza Filho et al., 2023), it is relevant to look upon the trend of these items over time (Fig. 9). They still show an increase once the festival returned, but at lower median counts.

Zip ties, although not classified as SUP under Directive 2019/904, represent a noteworthy item category within the dataset, as they are typically single-use. While they do not appear among the top 15 items in the CHILLOUT zone, their median abundances reach 33 items per 100 m in the VIP zone and 32 items per 100 m in the STAGE zone. Their presence is consistent with their common use in event logistics, including bundling and securing musical equipment during transport and setup, organizing electrical and audio cables, and stabilizing temporary structures such as protective barriers.

## Discussion

### Context-specific observations

Spatial variations in litter composition across the festival area reveal clear links between specific zones and the activities occurring within them. Several item categories show markedly higher abundances in the VIP and STAGE zones compared with the CHILLOUT zone, reflecting the operational, logistical, and behavioral dynamics unique to each area. Higher litter densities in zones with intense human activity, such as the STAGE zone, align with previous research showing crowd concentration strongly correlates with litter accumulation (Asensio-Montesinos et al., 2019; Garcés-Ordóñez et al., 2020; Grelaud & Ziveri, 2020). This pattern is likely driven by event-specific behaviors, including the tendency for attendees to discard items on the ground during performances or in crowded settings (Rangoni & Jager, 2017). Such behaviors vary across demographic groups, reflecting differences in anti-litter attitudes, willingness to act, and perceived accountability (Lev et al., 2023). Infrastructure also plays a key role, as the availability and accessibility of waste disposal facilities strongly influence the likelihood of improper disposal (Rangoni & Jager, 2017).

These spatial and behavioral patterns are clearly reflected in several item categories, beginning with cigarette butts. These are recognized as one of the most common forms of personal litter around the world (Curtis et al., 2017). In Portugal, for instance, cigarette butts were the most frequent marine litter item found on Funchal beaches (Madeira Island) (Bettencourt et al., 2023). In the SOMNII festival area, they are the most frequent item in the STAGE zone. Cigarette butt pollution can be mitigated through a combination of disposal infrastructure and behavioral interventions. Araújo and Costa (2021) emphasize the importance of providing appropriate disposal options, such as portable ashtrays, while Bettencourt et al. (2023) highlight the role of economic disincentives, including fees on cigarette packs and fines for improper disposal. Raising awareness about the composition and long degradation times of cigarette butts in marine environments is also essential (Bettencourt et al., 2023). Additional measures, such as designated smoking areas equipped with dedicated receptacles and behavioral prompts like signage or bin-top messaging, can further reduce accidental littering and support responsible disposal, directly addressing the behavioral patterns identified in this study.

SUP items in the SOMNII festival, including cutlery/trays/straws, crisp and sweet packets, lolly sticks, caps and lids, plastic cups and bottles, and bottle caps, are closely associated with food and drink consumption within specific festival activity areas. Their reduction can be supported through reusable alternatives and deposit-return schemes (DRS), which have proven effective in preventing mismanaged waste and promoting resource and energy recovery (Schuyler et al., 2018). While broader economic instruments such as taxes can reduce plastic use (Schuyler et al., 2018), event-level interventions, such as incentivizing participants to bring their own reusable containers or implementing DRS for cups and food packaging, are more directly actionable. Existing systems such as ReCup (ReCup 2026) and ECOBOX (Ecobox n.d.), and Tiffin boxes demonstrate the feasibility of reusable serviceware, and multifunctional utensils (e.g., sporks or compact polypropylene sets) offer additional low-waste alternatives (Robb & Murphy, 2019).

Zip ties constitute another item with marked spatial clustering, particularly in the VIP and STAGE zones, where equipment installation and cable management are concentrated. Their prevalence reflects logistical practices specific to festival setups, such as the transportation, installation, and organization of equipment, and contrasts with urban beach studies where such items are less frequently reported (Fernández García et al., 2024; Fernández-Enríquez et al., 2024). Effective mitigation requires upstream planning, including replacing single-use plastic ties with reusable options (e.g., velcro for cable bundling, silicone or stainless steel for heavy-duty equipment) or biodegradable, plastic-free alternatives (e.g., cellulose-based ties) (Strudwick et al., 2024). Procurement rules prohibiting single-use fastening systems, combined with mandatory collection protocols during setup and teardown, such as issuing technicians a fixed number of ties and requiring their return, supported by portable collection buckets, can substantially reduce accidental loss. Training technical teams and conducting post-event audits further support compliance and help minimize this operationally driven litter stream.

Promotional materials are another category strongly linked to festival-specific practices, particularly brand-marketing strategies involving the distribution of branded cups, wristbands, and other items. Their reduction depends on upstream design and controlled distribution (Robb & Murphy, 2019). Eliminating unnecessary single-use promotional items, shifting to digital alternatives, and standardizing essential materials in durable or reusable formats can prevent most of this waste from entering the beach environment. When physical items are unavoidable, organizers can require recyclable mono-material designs and avoid open giveaways, which are strongly associated with littering. Dedicated collection points in high-traffic zones, combined with staff training and post-event audits, help minimize leakage and reinforce responsible disposal.

The festival-specific litter patterns identified above highlight several priority areas where SOMNII organizers can meaningfully reduce their environmental footprint. The concentration of cigarette butts in audience zones reveals the need to strengthen smoking-related management strategies, whether through improved disposal infrastructure, designated areas, or other behavioral interventions. The prominence of SUP items associated with food and drink consumption points to opportunities for expanding reusable systems, redesigning serviceware, or refining deposit-return mechanisms. The clustering of zip ties in technical areas reveals an operational source of single-use plastics that could be addressed through procurement choices, reusable fastening systems, or improved collection protocols. Finally, the presence of promotional materials reflects upstream design and distribution decisions that organizers can reassess to minimize unnecessary single-use items. Taken together, these insights illustrate how event-specific litter data can guide targeted, context-appropriate actions that support more sustainable and lower-impact festival operations.

Examples from other large events demonstrate the effectiveness of some of the abovementioned approaches. When organizers control the design, material composition, and distribution of single-use items, integrate circular-economy solutions, and implement targeted collection systems and DRS, waste volumes decrease and recovery rates improve (Martinho et al., 2018; Robb & Murphy, 2019). For instance, in 2019 the Glastonbury Festival eliminated single-use plastic bottles (Glastonbury Festival, 2019) and has since recycled more than 180 tonnes of waste (Binit, 2025). That same year, several Danish festivals partnered with Tuborg to introduce washable plastic glasses (Food Nation. Solutions of Tomorrow by Denmark, 2019), and the Roskilde Festival achieved a 92% return rate for reusable cups (World Economic Forum, 2019). In 2024, Roskilde also repurposed waste from previous editions into functional furniture (Roskilde Festival, 2025; SMALLRevolution, 2025). Together, these examples illustrate how targeted design and operational choices can substantially reduce event-related waste. In this context, the festival-specific litter patterns identified in our dataset provide concrete evidence to support the redesign of materials, the reduction of unnecessary single-use items, and the implementation of closed-loop systems that collectively reduce the environmental footprint of large coastal events.

### Alignment with regional and global patterns of beach litter composition and accumulation

The number of items recorded in the study area is high and far exceeds the European Threshold Value (EU TV) of 20 items/100 m (van Loon et al., 2020). This level of contamination cannot be attributed to the SOMNII festival alone, as the median density of SOMNII-related items (94 items/100 m) is substantially lower than that of items from OTHER sources (235 items/100 m). This pattern is consistent with the OSPAR dataset analysis by Andriolo and Gonçalves (2025), who reported that beaches in the Central region of Portugal, where the SOMNII festival is held, have historically exhibited some of the highest pollution levels along the North Atlantic Iberian coast (2002–2020). Regarding litter composition, the predominance of artificial polymer materials aligns with global evidence identifying plastics as the dominant marine pollutant (Ali & Shams, 2015; Fernández-Enríquez et al., 2024; Garcés-Ordóñez et al., 2020; Pervez et al., 2020). The high proportion of plastics (> 90%) also mirrors the values reported by Andriolo and Gonçalves (2024) for European beaches.

Spatial patterns further reflect known ecological processes. The accumulation of artificial polymer items in zones away from the shoreline and near dune vegetation supports previous findings that dune vegetation acts as an effective sink for marine litter. In Mediterranean coastal habitats, for example, Gallitelli et al. (2023) observed that dune plants predominantly trap macrolitter at the edges of vegetation patches. Conversely, the concentration of maritime-related items in the CHILLOUT zone, closer to the shoreline, is consistent with studies showing that fishing-related litter items tend to accumulate near the swash zone (Ouyang & Yang, 2022; Watson et al., 2022). However, this differs from earlier Portuguese studies reporting higher litter loads along the high tide line (Anastácio et al., 2023), suggesting that SOMNII may influence spatial distribution by concentrating festival attendees, and therefore waste, away from the shoreline.

Temporal patterns also reveal meaningful dynamics. Although the overall trend aligns with global tendencies, the seasonal peak in summer contrasts with typical patterns on the North Atlantic Iberian Coast, where higher litter loads are usually recorded in autumn and winter. In this case, the summer maximum is consistent with increased tourist presence (Asensio-Montesinos et al., 2019; Garcés-Ordóñez et al., 2020) and with the documented impact of large-scale events on coastal pollution (Oliveira et al., 2022). The sharp reduction in litter during festival-free years (2020–2021) reflects the effect of COVID-19 lockdowns in reducing terrestrial inputs (Currie et al., 2023; Souza Filho et al., 2023). The slight decrease in 2023, when a massive cleanup regime was implemented after the festival, aligns with evidence on the effectiveness of targeted waste management strategies (Rangoni & Jager, 2017). Together, these temporal variations reinforce the importance of long-term monitoring, which enables the detection of trends that short-term studies may overlook. In the particular case of this study, the observed fluctuations point to the combined influence of pandemic-related restrictions, event-driven pressures, and cleanup interventions. More broadly, such datasets are essential for evaluating the effectiveness of environmental policies, public awareness campaigns (Dodds et al., 2022; Gration et al., 2011; Williams & Rangel-Buitrago, 2019), and mitigation strategies for large coastal gatherings (Gallagher & Pike, 2011; Oliveira et al., 2022).

### Advancing monitoring methodologies: a stratified OSPAR framework for event-driven litter dynamics

Despite increasing recognition of beaches as critical monitoring sites for marine litter, standardized methodologies, including OSPAR’s, remain optimized for long-term, low-frequency monitoring across broad spatial units (Wenneker et al., 2010). These designs struggle to capture locally important litter items (Falk-Andersson, 2021; Falk-Andersson et al., 2025), as well as acute, short-lived pollution events associated with high-density, high-tempo activities like beach festivals (Gallagher & Pike, 2011). This study addresses these methodological blind spots by adapting the OSPAR monitoring protocol into a stratified, high-resolution sampling framework tailored to the spatiotemporal dynamics of a large-scale beach event, capable of capturing litter items specifically related to the SOMNII event.

By partitioning the SOMNII festival site into three functionally distinct zones—VIP, STAGE, and CHILLOUT—each sampled using fixed 100 m × 50 m plots, this approach enabled fine-scale detection of litter accumulation and composition. Stratification revealed significant spatial differences: STAGE areas averaged around seven times more litter than CHILLOUT zones, and litter composition varied by zone and source. Without this zoning, such detail would likely have been obscured under a conventional 100-m transect model (Asensio-Montesinos et al., 2019). Importantly, the method uncovered clear temporal shifts in litter dynamics, driven by external events such as COVID-19-related festival cancellations and the introduction of intensive post-festival cleanups in 2023.

The results indicate that stratified OSPAR monitoring not only enhances the sensitivity of event-driven litter detection but also improves hotspot identification, informing better spatial targeting of waste prevention measures (van Loon et al., 2020). These insights offer a scalable monitoring template for other tourist-intensive coastal environments, where functional zoning (e.g., event spaces, recreational zones, food/beverage areas) can vary but follow broadly similar patterns (Dodds et al., 2022). Event organizers and coastal managers may adopt this framework to create “event-specific litter response plans,” combining real-time monitoring with targeted interventions such as bin placement, signage, and reusable item programs (Prasuhn et al., 2017).

While the approach is broadly applicable and logistically feasible, as it leverages standard OSPAR analysis protocols and tools (e.g., the R package litterR), it is not without limitations. Zone-specific monitoring requires coordination with event planners and local authorities to ensure access and optimal timing, and although not more labor-intensive than traditional approaches, increased temporal frequency may impact resource needs. The classification of items by source, while grounded in site-specific observations and item characteristics, also carries some uncertainty, particularly for unbranded plastic fragments such as string and cord (diameter less than 1 cm), and cross-purpose items such as zip ties (van Loon et al., 2020). In addition, the transferability of the zonation scheme depends on the spatial configuration of other events, meaning that direct replication is not always feasible. These constraints also limit the suitability of the method for real-time decision-making during fast-paced events.

Despite the limitations, the insights gained from the stratified approach justify its added complexity, offering a replicable and scientifically robust enhancement to conventional marine litter monitoring (Bravo et al., 2021; Ramos et al., 2016). By integrating functional spatial analysis into existing frameworks, this method supports both regulatory evaluation (e.g., OSPAR’s RAP ML and the EU SUP Directive (Directive 2019/904)) and the design of targeted, evidence-based waste mitigation strategies (Gallagher & Pike, 2011).

For operational contexts where rapid responses are required, a complementary rapid-assessment approach can help bridge this gap. In the case of SOMNII, this could involve short, high-frequency visual scans of predefined high-risk zones (e.g., STAGE front, VIP access corridors, food and beverage areas), combined with simple tally sheets focused on the most abundant or problematic categories identified in this study, such as cigarette butts, SUP food-service items, and zip ties. While these rapid assessments would not replace stratified monitoring, they would provide organizers with near-immediate feedback, enabling timely adjustments to bin placement, staff deployment, signage, or distribution practices. Integrating rapid assessments with more detailed post-event stratified surveys therefore offers a balanced framework: real-time operational responsiveness supported by robust, spatially explicit evidence for long-term planning.

### Policy leverage and collaborative strategies for event-driven litter mitigation

Large-scale coastal events present a valuable opportunity to evaluate the effectiveness of mitigation efforts and shape future waste management strategies. Following the 2023 edition of the RFM SOMNII festival, extensive cleanup operations led to marked reductions in litter density, particularly in high-traffic areas. This decrease, most pronounced in the STAGE and VIP zones, highlights the effectiveness of targeted cleanup interventions in reducing the environmental footprint of such events (Rangoni & Jager, 2017). At the same time, the high prevalence of artificial polymer fragments reveals the limitations of cleanup operations, which primarily address visible waste while overlooking less conspicuous pollutants (Galgani et al., 2015; Santillán et al., 2023). Plastic fragments are of particular concern because they often exhibit inherent weaknesses that make them more susceptible to degradation (Baby et al., 2024). While both large and small items pose environmental risks, smaller fragments are especially problematic because they are more difficult to detect and remove during cleanup, increasing the likelihood that they remain in the environment and elevating long-term management costs. Their persistence also heightens biological risks, as these particles are ingested by marine organisms and can enter the food chain, with potential consequences for ecosystem health and human well-being (Browne et al., 2015). Moreover, the unidentifiable nature of litter fragments (Zielinski et al., 2022) raises litter categorization issues that skew conclusions (Watts et al., 2017) and make the implementation of targeted measures more challenging. In the context of litter monitoring events, it also exacerbates the problem of distinguishing between event-related waste and pre-existing litter. While branded items like cups or wristbands can be directly linked to an event, a significant portion of the litter consists of non-branded or fragmented items that are harder to trace. To overcome these constraints, Falk-Andersson (2021) suggests the implementation of complementary, adaptive and participatory methodologies, named “Beach Litter Deep Dives,” to get a better understanding of the sources of and behavior behind littering.

The findings provide empirical support for the implementation and enhancement of policies such as the EU Single-Use Plastics (SUP) Directive (Directive 2019/904) and the OSPAR Regional Action Plan on Marine Litter (RAP ML 2) 2022–2030 (OSPAR Commission, 2022). These frameworks aim to reduce plastic pollution through measures such as the restriction or elimination of SUP items, implementation of the polluter pays principle, improved waste management, and awareness raising.

SUP, including plastic cups, straws, and cutlery, were banned in the European Union in July 2021 (Directive 2019/904). The SOMNII festival appears to reflect the early success of these policies, as the presence of these items decreased in 2022 and 2023 compared with the 2019 edition. However, some banned items, and other plastic items such as cigarette butts and food-related packaging, still persisted, particularly in high-density areas and despite the cleaning efforts. Implementing event-specific policies, such as extending bans to additional items or mandating reusable alternatives, could help address these gaps (Williams & Rangel-Buitrago, 2019). Notably, during the SOMNIII festival, single-use plastic cups were replaced with reusable plastic cups, yet this substitution also presents challenges: many cups still ended up on the beach, sometimes fragmented, and it remains unclear whether these reusable plastic items contained chemicals that could pose health risks (Al-Mansoori et al., 2025; Hussain et al., 2023; Tisler et al., 2024). Other potential alternatives to SUPs were not observed during the SOMNII monitoring, but careful evaluation is needed to ensure that banned items are replaced with genuinely sustainable options rather than single-use products made from different materials (e.g., a wooden cutlery, which may degrade faster but can still harm wildlife). It is therefore crucial to include replacement items in the OSPAR protocol to ensure they do not go unrecorded, which would otherwise create an additional monitoring limitation. The establishment of Extended Producer Responsibility (EPR) schemes for packaging (Directive 2019/904) should support this process by facilitating the setting and monitoring of reusable targets. Based on the polluter pays principle, EPR schemes require manufacturers and importers to take responsibility for the environmental and social costs of their products throughout the product lifecycle, covering costs of collection, treatment, management, clean up, and awareness raising. This should encourage festival organizers to focus not only on downstream impacts from the use and disposal of products, by collaborating with manufacturers to rethink product design and setting effective collection schemes (e.g. DRS), but also on upstream impacts, by motivating manufacturers to reconsider material selection and adapt the production processes. However, the effectiveness of EPR schemes faces several constraints. Implementation and enforcement remain uneven across Member States (European Commission, 2025), which can weaken incentives for genuine eco-design. In many systems, fee structures still prioritize recycling over reuse, offering limited differentiation to drive shifts toward durable or low-impact materials (Linder et al., 2017; Maitre-Ekern, 2021; Micheaux & Aggeri, 2021). Smaller manufacturers and local festival suppliers may also encounter administrative and financial burdens that slow adaptation (Battay et al., 2025). Moreover, EPR, as mandated by the SUP Directive (Directive 2019/904), does not fully cover some festival-relevant items, such as promotional materials or operational consumables (Directive 2019/904), leaving gaps in responsibility allocation. These limitations mean that, while EPR can support upstream and downstream improvements, its impact ultimately depends on robust implementation and complementary measures adopted by organizers and suppliers.

While challenges related to production, consumption reduction, and product redesign are critical, effective waste management and public awareness remain central to addressing the litter crisis. Waste management practices implemented at large-scale events in Portugal (Interreg Europe, 2022) and Málaga (Interreg Europe, 2025) demonstrate the positive outcomes of such measures. Stricter regulations for event organizers could include requirements for comprehensive cleanup plans (Rangoni & Jager, 2017; Williams & Rangel-Buitrago, 2019). Rangoni and Jager (2017), for instance, recommend adaptive/dynamical cleaning regimes, which appear to be more effective and cost-efficient than predefined schedules. Such approaches may also mitigate issues that arise during peak festival hours, when crowd movement can disperse litter into adjacent areas, complicating collection efforts and reducing cleanup efficiency. Additional sustainable practices for large-scale events could include expanding recycling opportunities through the use of smart bins and AI-driven waste sorting systems, which can improve the efficiency of waste segregation and processing (Ramasawmy & Nagowah, 2023).

Public awareness campaigns are another essential component in addressing the pollution crisis, particularly when they foster behavioral changes that lead to long-term reductions in litter generation (Bär et al., 2022). Their importance is amplified by the fact that many individuals still lack basic knowledge about the degradability of materials, appropriate disposal practices, and the environmental footprint associated with different products (Escobar-Sánchez et al., 2025; Nejadsadeghi et al., 2025). Recent behavioral-science research shows that well-designed communication interventions can significantly influence waste-related decisions by addressing key psychological determinants of littering (Hansmann & Steimer, 2015; Mori et al., 2024; Ojedokun & Balogun, 2013; Tobias et al., 2024). For festival settings, a combination of digital platforms and in-person initiatives can help build a sense of shared responsibility among attendees. Clear signage supported by intuitive, visual instructions on waste segregation is one effective approach, and studies show that such cues can reduce littering when they are salient and context-specific (Hansmann & Steimer, 2015). Interactive strategies, including reward systems for proper waste sorting, gamified challenges, or “green camp” recognition schemes, can further enhance engagement and incentivize responsible behavior (Chen et al., 2024; Hu et al., 2025). Additional evidence-based measures include social-norm messaging, which leverages peer expectations to reduce littering (Ojedokun & Balogun, 2013), and face-to-face outreach, which has been shown to strengthen pro-environmental intentions when combined with visual prompts (Hansmann & Steimer, 2015). Together, these approaches illustrate how targeted awareness initiatives can complement infrastructural and regulatory measures to reduce litter generation at large events.

Examples from Roskilde Festival illustrate how such strategies can be embedded in festival culture: community camping concepts like “Leave No Trace” and “Clean Out Loud” explicitly communicate expected behavior and engage participants in shared responsibility for campsite cleanliness, contributing to substantial reductions in waste left behind (Future Festival Tools. Green Competency for Festival Professionals, 2022). In turn, DGTL festival, which aimed to become the first circular, climate neutral major event, is on its journey to find ways to change the linear behavior of visitors to a circular behavior, while explaining to its visitors what happens to the materials during and after the festival and shows them how and why it happens to make visitors aware of environmental issues (Future Festival Tools. Green Competency for Festival Professionals, 2022). Both Roskilde and DGTL frame sustainability as a collective effort, encouraging attendees to sort their waste, reuse gear, and make low-impact choices as part of a broader movement toward a “smaller footprint” (Future Festival Tools. Green Competency for Festival Professionals, 2022). Alcohol consumption adds an additional challenge, as it can reduce self-control and weaken adherence to social norms, including those related to littering. Some festivals have begun addressing this indirectly through responsible-drinking initiatives. For example, Roskilde Festival has partnered with Carlsberg to promote low- and zero-alcohol options, aiming to support more responsible behavior throughout the event (Carlsberg Group, 2024). While such measures may help reinforce pro-environmental norms, empirical evidence directly linking alcohol-reduction strategies to litter outcomes remains limited, highlighting the need for further research on how intoxication influences waste-related behavior in festival settings.

The findings point to the necessity of proactive collaboration of event organizers with researchers, industry, and local decision-makers. Collaboration with researchers would support the identification and tracking of specific items more efficiently (Zielinski et al., 2022). This could support the identification of potential waste hotspots within the event site, helping organizers to implement targeted waste collection strategies (e.g., strategically placing bins or staff deployment), translated into cost savings. It could identify problematic waste sources, enabling organizers to adopt sustainable practices and comply with environmental regulations, such as reducing single-use plastics or promoting reusable alternatives. It could also provide valuable data on attendee behavior and waste generation patterns, which can inform future event planning and layout optimization (Martinho et al., 2018). One collaboration approach could involve authorizations for pre- and post-event litter composition surveys to establish clearer baselines for monitoring the effectiveness of interventions over time (Zielinski et al., 2022). Another collaboration could involve tagging branded items distributed during the events with advanced technologies such as QR codes (Aparna et al., 2022), enabling researchers to trace the origins of specific items more efficiently. This would also need to rely on collaboration with industry to provide such technologies. Other collaborations with industry could focus on developing and adopting alternatives for problematic items, such as zip ties, for which sustainable options, on paper, bio-based plastics, and recycled PET are already available. Some festival organizers, such as those of the Body & Soul Festival in Ireland (Future Festival Tools. Green Competency for Festival Professionals, 2022), report that it is more cost-efficient to collaborate with sustainable event suppliers because designing and understanding the infrastructure needed to make a festival more sustainable is highly challenging. However, they also note that Ireland has very little infrastructure and expertise to support sustainability at festivals. This may also pose a limitation for other events that aim to source materials and services locally as a way to enhance their sustainability, a point supported by research when considering all five dimensions of sustainability (environmental, economic, social, health, and ethics) into consideration (Schmitt et al., 2017).

Collaboration with decision-makers plays a pivotal role in implementing targeted solutions for environmental sustainability at events. Such partnerships can take various forms. For example, event organizers and decision-makers can coordinate the installation of adequate waste management infrastructures, such as recycling bins, composting stations, and waste collection points, to address the unique challenges of coastal environments (Oliveira et al., 2022). Joint efforts can also be directed toward public awareness campaigns that educate attendees on the environmental impacts of littering and foster responsible waste disposal behaviors (Mair & Laing, 2013). Furthermore, collaborative initiatives can extend to reward systems, such as discounts or tokens, to encourage attendees to engage in proper waste segregation and recycling (Yang et al., 2024). Additionally, local authorities can play a key role in establishing or promoting adherence to sustainability certifications for events, which serve as benchmarks for effective waste management and environmental responsibility (Nygaard, 2023) and can also incentivize the redirection of reusable or repurposable waste through the implementation of donation programs benefiting local institutions (Oliveira et al., 2022).

## Conclusions

This study demonstrates the value of adapting established monitoring frameworks, specifically the OSPAR methodology, to better capture the spatial and temporal complexities of marine litter generated during large-scale coastal events. By implementing a stratified sampling design across functionally distinct zones of the RFM SOMNII festival site, the research revealed clear patterns of litter accumulation and material composition that would likely have been missed using conventional beach monitoring protocols. These insights not only advance our understanding of event-driven pollution dynamics but also highlight the potential of tailored methodologies to inform more targeted and effective waste management interventions.

While the dominance of artificial polymer materials and single-use plastics (SUP) remains a consistent challenge, the study demonstrates that refined sampling approaches can support hotspot detection and improve attribution. These are critical steps for prevention and mitigation. The unique context of the COVID-19 pandemic further enabled a contrast between festival-active and inactive periods, providing rare empirical evidence on the direct influence of human activity.

Beyond the case study, the stratified monitoring framework offers a replicable blueprint for enhancing marine litter assessments under similarly dynamic and high-pressure coastal conditions. Its application can improve decision-making by identifying pollution hotspots, refining litter source analysis, and supporting compliance with policy frameworks such as the EU SUP Directive (Directive 2019/904) and OSPAR’s Regional Action Plan. Future research should explore how integrating traceability technologies, automated detection tools, and stakeholder collaboration can further strengthen attribution accuracy and operational efficiency. Together, these innovations can accelerate progress toward more sustainable coastal event management and marine environmental protection.

## Supplementary Information

Below is the link to the electronic supplementary material.

ESM 1 (PDF 301 KB)

ESM 2 (PDF 2.10 MB)

## Data Availability

Data is available at https://zenodo.org/records/15303685 under the title "Dataset Seasonal Data on Beach Litter from a Massive Music Festival in Portugal (2019–2023)" (Anonymous). The dataset is available in raw and processed formats.
