# Supplementary material for: Stratifying the shoreline: a modified OSPAR framework to monitor event-driven beach litter
Source: Environ Monit Assess. 2026 Apr 10;198(5):433. doi: 10.1007/s10661-026-15260-x (PMC13068741; doi:10.1007/s10661-026-15260-x)
Supplement: Supplementary file 1 — (PDF 301 KB) [file 10661_2026_15260_MOESM1_ESM.pdf]

DOI: 10.1007/s10661-026-15260-x

Stratifying the Shoreline: A Modified OSPAR Framework to Monitor Event-Driven Beach Litter

Supplementary Material - Chi-square test

Litter categories relationship with other variables | Chi-square test

All categorical variables (category, source, sampling units, seasons, and years) exhibit statistically significant associations with litter categories ($p < 0.05$), indicating dependence among them (Table S1). Table S2 highlights, in bold, associations contributing over 10% to the total Chi-Square score, accounting for most of the difference between expected and observed values.

Table S1. Pearson's Chi-squared test of independence to evaluate the association between litter categories and the categorical variables source (SOMNII/OTHER), sampling unit (VIP, STAGE and CHILLOUT zones), sampling season (winter, spring, summer, autumn) and sampling year (2019, 2020, 2021, 2022, 2023).

	Chi-square value	df	p-value
**Litter categories VS Source**	4075.6	7	< 2.2e-16 *
**Litter categories VS Sampling Unit**	655.06	14	< 2.2e-16 *
**Litter categories VS Season**	1052.9	21	< 2.2e-16 *
**Litter categories VS Year**	1358.5	28	< 2.2e-16 *

* p-value <0.05 (statistically significant)

Table S2. Relative contribution (%) of each association to the total Chi-square score. In **bold** are contributions larger than 10%.

	Source		Sampling Units			Seasons				Years				
Category	OTHER	SOMNII	VIP	STAGE	CHILLOUT	autumn	spring	summer	winter	2019	2020	2021	2022	2023
Artificial Polymer Material	**-17.49**	**14.34**	-2.44	1.55	-0.17	-3.93	2.5	0.08	1.32	-4.95	0.29	-0.75	2.41	3.56
Cloth	**11.16**	-9.15	3.37	-2.33	0.89	1.63	-1.36	0.13	-0.52	0.33	0.1	2.42	0.85	-2.93
Glass & ceramics	**17.79**	**-14.59**	-0.85	1.15	-2.13	**19.57**	-0.52	**-10.24**	0.98	5.57	6.32	-1.03	-4.09	-7.05
Metal	**13.61**	**-11.16**	-3.93	2.01	1.38	**12.39**	-2.18	-6.43	2.89	0.2	**10.97**	-2.75	-2.73	-5.03
Paper/Cardboard	**27.28**	**-22.37**	**18.37**	**-12.17**	2.93	0.94	-0.36	1.47	-4.11	0.34	-6.16	8.43	0.42	-1.22
Processed/ Work.Wood	**25.67**	**-21.05**	-7.29	5.47	-3.36	-7.31	-9.52	11.09	-4.8	**24.18**	-7.88	-5.92	-8.93	-8.26
Rubber	5.76	-4.73	-1.65	1.32	-1.06	1.99	-2.72	1.09	-1.46	-2.09	-2.04	-0.82	0.6	4.09
Undefined	8.28	-6.79	1.58	-1.46	1.67	0.97	0.98	-1.04	-0.01	-0.99	-1.56	5.42	-0.4	-0.8
