# Supplementary material for: Stratifying the shoreline: a modified OSPAR framework to monitor event-driven beach litter
Source: Environ Monit Assess. 2026 Apr 10;198(5):433. doi: 10.1007/s10661-026-15260-x (PMC13068741; doi:10.1007/s10661-026-15260-x)
Supplement: Supplementary file 2 — (PDF 2.10 MB) [file 10661_2026_15260_MOESM2_ESM.pdf]

DOI: 10.1007/s10661-026-15260-x

Stratifying the Shoreline: A Modified OSPAR Framework to Monitor Event-Driven Beach Litter

Supplementary Material – Images

This document presents five groups of images:

1. Official maps of the festival grounds.
2. Images of festival zones, taken during the SOMNII 2019 festival.
3. Images of items left behind by festival participants or staff, taken during the SOMNII 2019 festival.
4. Images taken during the field sampling process.
5. Images of items found during the sorting process.

1. Official maps of the festival grounds

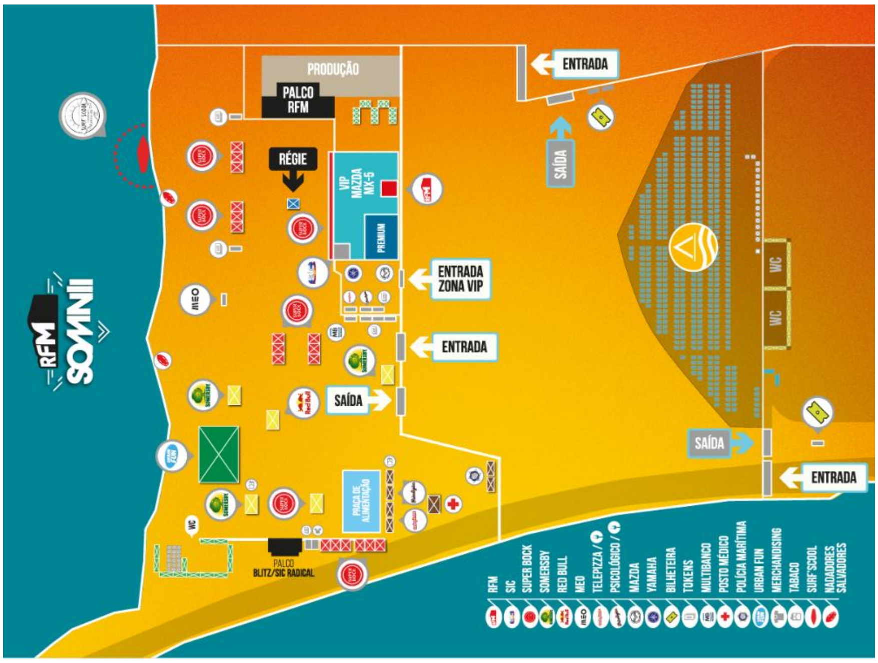

Figure S1. Official map of SOMNII 2019 edition, immediately before the first sampling campaign. The image has been horizontally flipped so that it is oriented to the north, with the Atlantic Ocean shown on the left.

DOI: 10.1007/s10661-026-15260-x

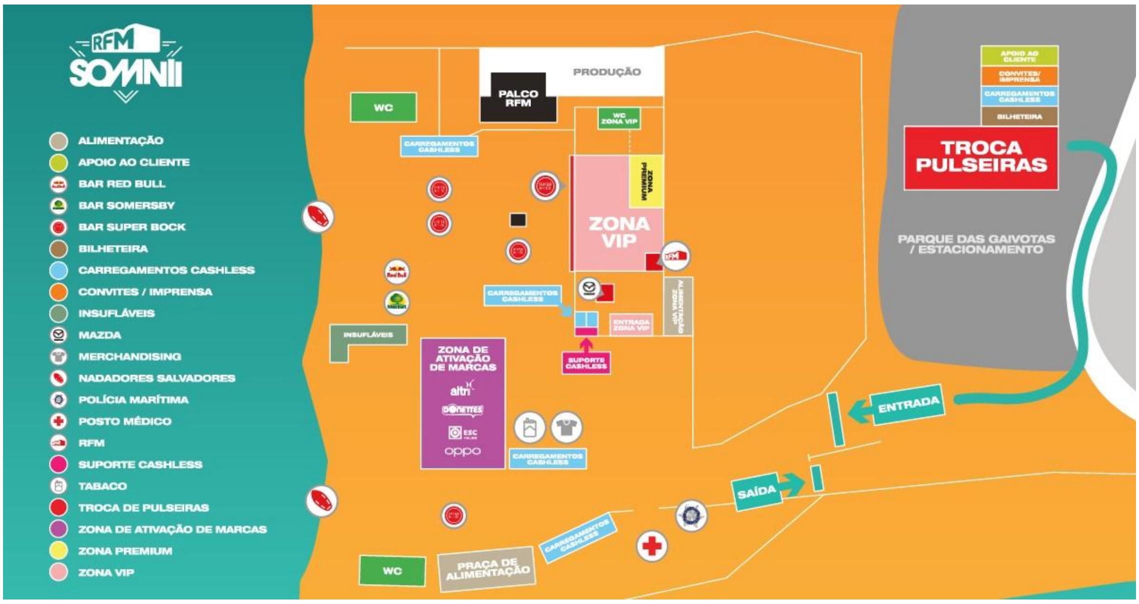

Figure S2. Official map of SOMNII 2023 edition, immediately before the last sampling campaign.

2. Images of festival zones

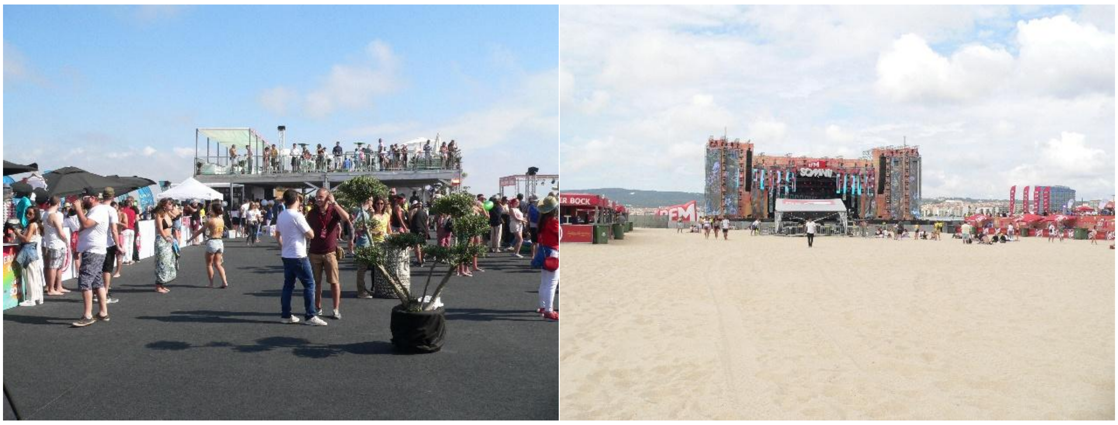

Figure S3. Left: VIP zone in 2019, featuring a carpeted floor. **Right**: STAGE zone in 2019, during the day.

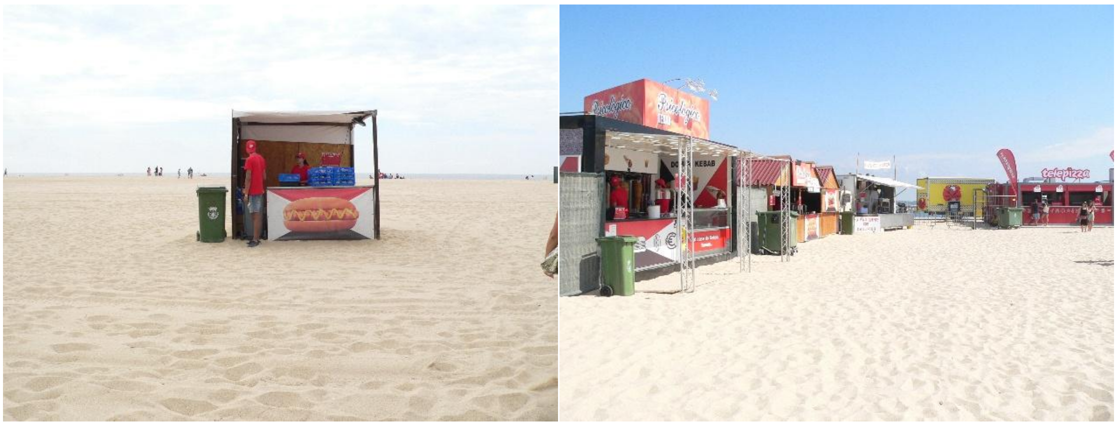

Figure S4. Left: Small food tent with CHILLOUT zone behind, in 2019. **Right**: Food court in 2019. Outside the sampling area.

3. Example of items left behind by festival participants or staff

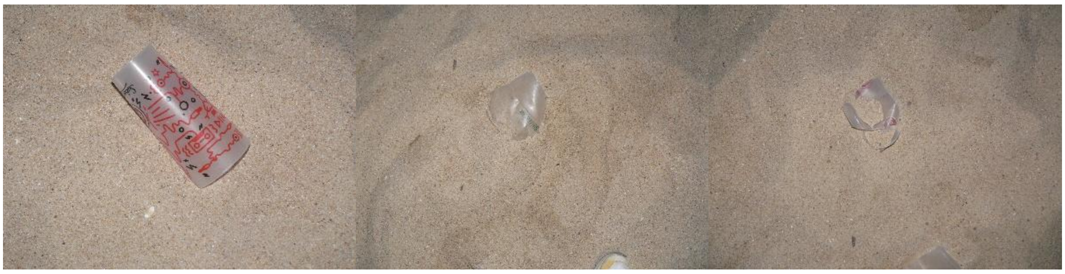

Figure S5. Single-use plastic cups.

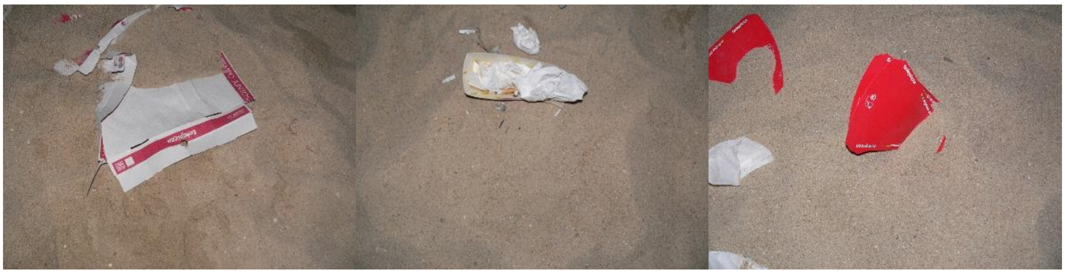

Figure S6. Single-use cardboard food containers.

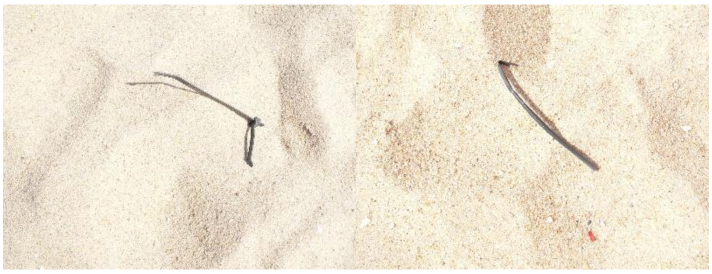

Figure S7. Zip ties.

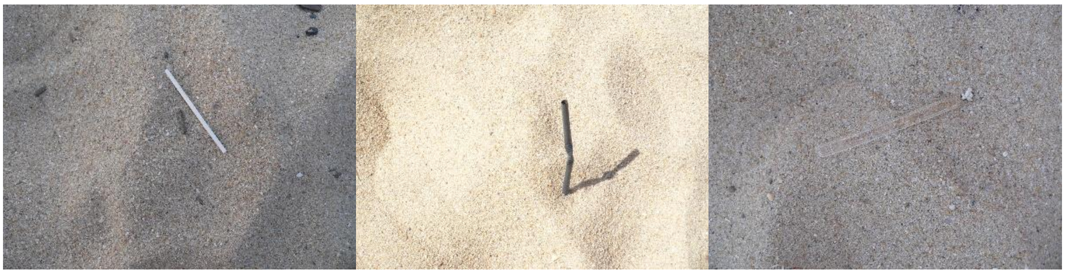

Figure S8. Left and Middle: Plastic straws. Right: Plastic coffee stirrer.

DOI: 10.1007/s10661-026-15260-x

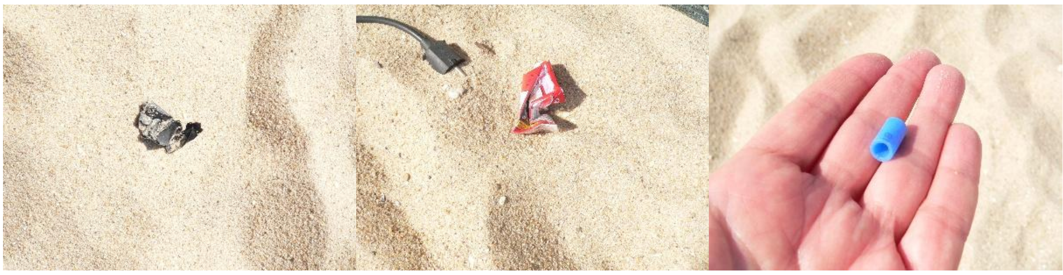

Figure S9. Left: Plastic adhesive tape. Middle and right: Electric appliances.

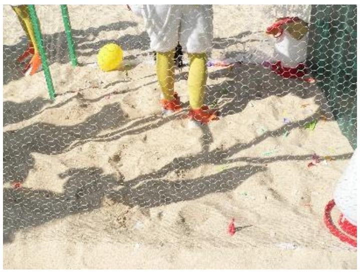

Figure S10. Balloons and burst balloons, from an entertainment area.

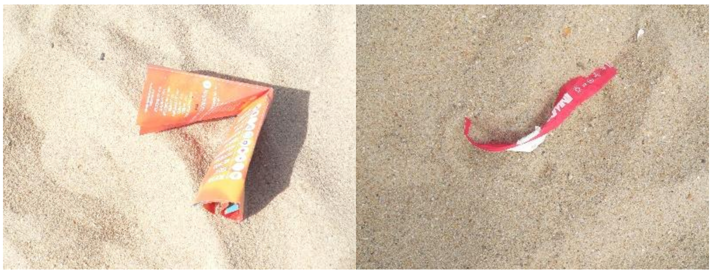

Figure S11. Left: Festival programme. Right: Festival Festival wristband

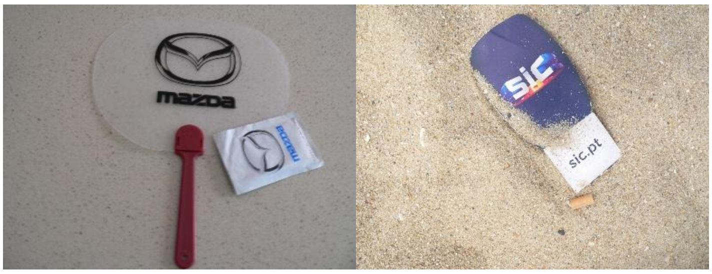

Figure S12. Merchandise from sponsoring brands.

4. **Images taken during field sampling process**

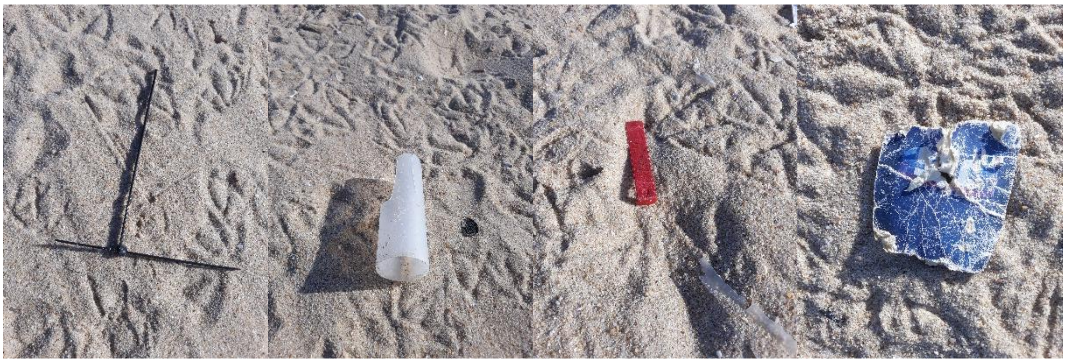

Figure S13. Examples of items found and collected during the Summer 2019 field campaign in the STAGE zone. From left to right: a zip tie, a plastic cup, a plastic keg seal, cardboard merchandise.

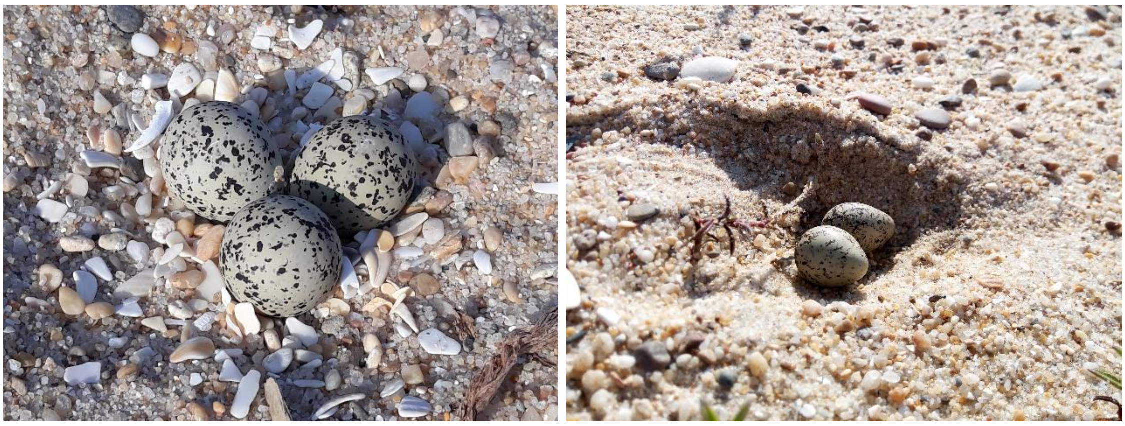

Figure S14. *Charadrius alexandrinus* eggs discovered in the VIP sampling zone during the Spring 2020 (left) and Spring 2021 (right) field campaigns (few weeks before the festival).

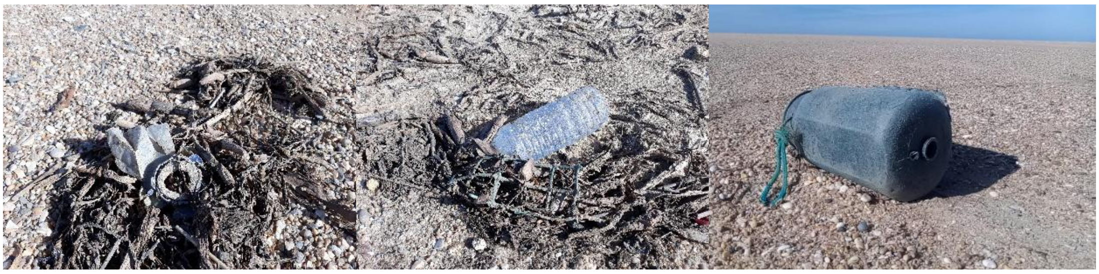

Figure S15. Examples of items found and collected during the Winter 2021 field campaign in the CHILLOUT zone.

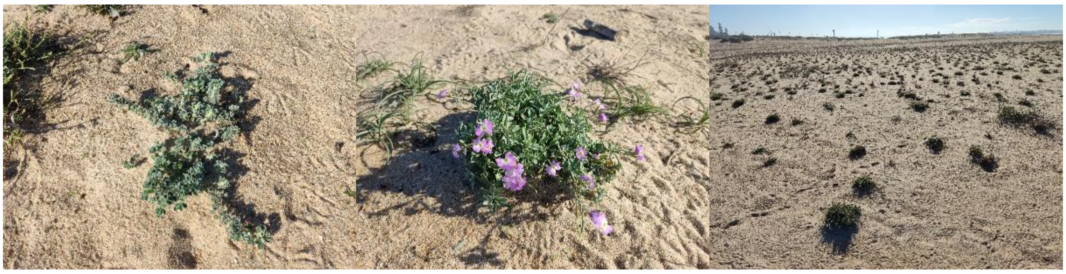

Figure S16. Dune vegetation thriving in the festival area during the years when the event was canceled due to COVID-19. Pictures taken during the Autumn 2022 field campaign.

DOI: 10.1007/s10661-026-15260-x

5. Items found during the sorting process

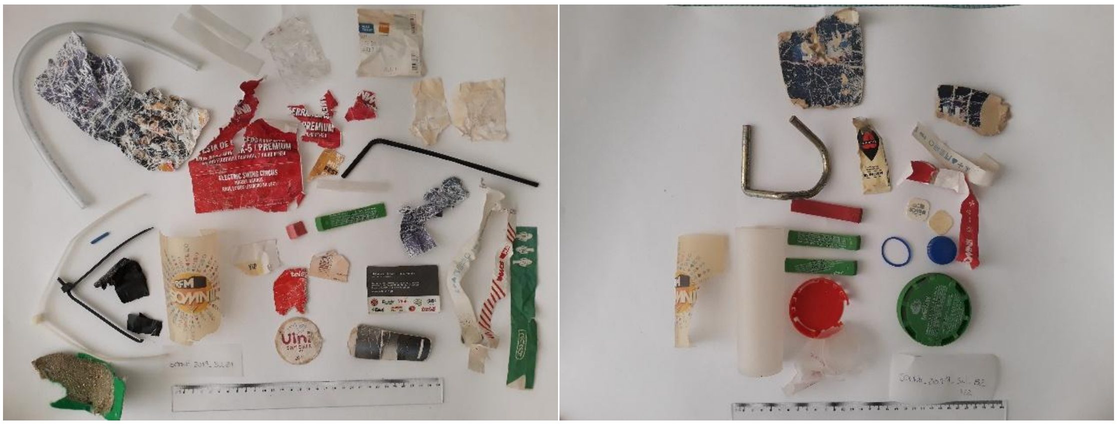

Figure S17. Example of items sorted from the Summer 2019 field campaign. Left: VIP Zone. Right: STAGE zone.

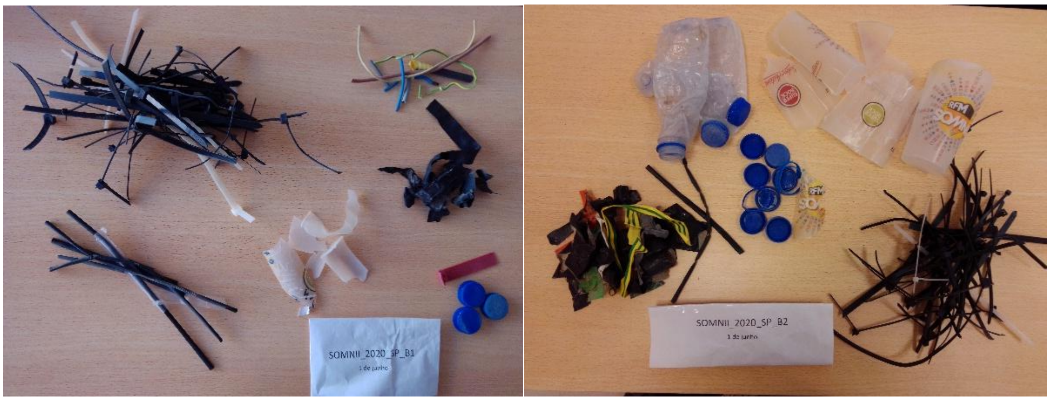

Figure S18. Example of items sorted from the Summer 2020 field campaign. Left: VIP Zone. Right: STAGE zone.
